# Delineating the Genetic Component of Gene Expression in Major Depression

**DOI:** 10.1016/j.biopsych.2020.09.010

**Published:** 2021-03-15

**Authors:** Lorenza Dall’Aglio, Cathryn M. Lewis, Oliver Pain

**Affiliations:** a Social, Genetic and Developmental Psychiatry Centre, Institute of Psychiatry, Psychology and Neuroscience, King's College London, London, United Kingdom; b Department of Medical and Molecular Genetics, Faculty of Life Sciences and Medicine, King’s College London, London, United Kingdom; c Department of Child and Adolescent Psychiatry, Erasmus University Medical Center Rotterdam, Rotterdam, The Netherlands; d Generation R Study Group, Erasmus University Medical Center Rotterdam, Rotterdam, The Netherlands

**Keywords:** Depression, Expression, Genes, Genetics, MD, TWAS

## Abstract

**Background:**

Major depression (MD) is determined by a multitude of factors including genetic risk variants that regulate gene expression. We examined the genetic component of gene expression in MD by performing a transcriptome-wide association study (TWAS), inferring gene expression–trait relationships from genetic, transcriptomic, and phenotypic information.

**Methods:**

Genes differentially expressed in depression were identified with the TWAS FUSION method, based on summary statistics from the largest genome-wide association analysis of MD (*n* = 135,458 cases, *n* = 344,901 controls) and gene expression levels from 21 tissue datasets (brain; blood; thyroid, adrenal, and pituitary glands). Follow-up analyses were performed to extensively characterize the identified associations: colocalization, conditional, and fine-mapping analyses together with TWAS-based pathway investigations.

**Results:**

Transcriptome-wide significant differences between cases and controls were found at 94 genes, approximately half of which were novel. Of the 94 significant genes, 6 represented strong, colocalized, and potentially causal associations with depression. Such high-confidence associations include *NEGR1, CTC-467M3.3, TMEM106B, LRFN5, ESR2,* and *PROX2*. Lastly, TWAS-based enrichment analysis highlighted dysregulation of gene sets for, among others, neuronal and synaptic processes.

**Conclusions:**

This study sheds further light on the genetic component of gene expression in depression by characterizing the identified associations, unraveling novel risk genes, and determining which associations are congruent with a causal model. These findings can be used as a resource for prioritizing and designing subsequent functional studies of MD.

SEE COMMENTARY ON PAGE e31

Major depression (MD) constitutes one of the largest contributors to global disability worldwide, affecting around 322 million people and accounting for approximately 50 million years lived with disability (1). This disorder is of complex origin, being determined by the interplay of a multitude of environmental factors (e.g., life events) and genetic variations (2). The genetic contribution to MD has been shown by twin studies, which yielded heritability estimates of approximately 37% (3), and genome-wide association studies (GWASs), which estimated a 9% heritability as captured by common single nucleotide polymorphisms (SNPs) (4).

GWASs have started to identify the specific genes underlying depression genetic risk. In one of the most recent and largest GWASs to date (*n* = 135,458 cases, *n* = 344,901 controls), Wray *et al.* (5) uncovered 44 independent loci associated with MD. Drawing on a larger sample (*n* = 246,363 cases, *n* = 561,190 controls) and a broader phenotype definition, Howard *et al.* (4) observed 102 independent loci, further demonstrating the polygenicity of the disorder. While GWASs have enabled the identification of SNPs conferring susceptibility to depression, the functional significance of such genetic variants remains to be elucidated. The examination of intermediary processes between genomics and the phenotype, such as gene expression, would permit greater insight into the molecular mechanisms underlying depression.

Gene expression is a biological process permitting the translation of the genetic code into functional products, such as proteins and functional RNA. While gene expression is affected by extrinsic exposures, a substantial role for SNPs on the transcriptome has also been observed, with 50% of common variants being associated with gene expression, in any tissue (6). Genetic variants affecting gene expression are referred to as expression quantitative trait loci (eQTL).

Studies mapping eQTLs enabled the development of publicly available reference panels containing information on SNP-transcription relationships, in multiple tissues, across different samples. Based on these, gene expression can be predicted in any genetically mapped trait. The use of predicted gene expression allows for the analysis of transcription-trait associations with larger samples, inexpensively, and in multiple tissues, as opposed to classical observed gene expression studies. Importantly, such a novel approach solely captures the genetic component of gene expression, i.e., gene expression resulting from SNP variation (usually in *cis*, within 500 kb from gene boundaries). Differential expression that is a consequence of depression (reverse causation) can, therefore, be excluded.

Several methods to predict transcription-trait associations are available. These leverage 3 key sources of information: 1) reference panels with data on SNPs-transcription associations (e.g., GTEx [Genotype-Tissue Expression] Consortium), 2) individual- or summary statistic–level GWAS data capturing SNP-trait associations, and 3) a linkage disequilibrium (LD) (i.e., correlations across SNPs) reference. Of the available methods, transcriptome-wide association studies (TWASs) (7,8) are the most commonly used. These explore the transcription-trait association with a gene-by-gene approach. The integration of SNP effects into genes lowers the multiple testing burden and increases power, thus allowing TWASs to identify genetic associations potentially overlooked by GWASs (8).

To date, 4 studies have tested for an association between the genetic component of gene expression and depression (5,9, 10, 11). Wray *et al.* (5) showed transcriptomic differences in MD at 17 genes in the dorsolateral prefrontal cortex (DLPFC). Other studies extended these findings by identifying a greater number of associations, for MD (9,10) as well as broad depression (11), across multiple tissues (i.e., distinct brain tissues and whole blood).

Yet, no TWAS to date has extensively examined these associations. This impeded the identification of which transcriptomic changes are most relevant to depression. As genes correlate in their expression and LD can determine the detection of noncausal genes in TWASs (12), it is important to better delineate the genetic component of gene expression. Moreover, previous studies generally examined brain and blood tissues only, thus overlooking the role of the hypothalamic-pituitary-adrenal (HPA) and hypothalamic-pituitary-thyroid (HPT) axes. These are important in MD owing to their involvement in the hypo- and hyperactivity of stress responses, sleeping difficulties, and weight loss—all physiological dysfunctions shown in patients with depression (13, 14, 15).

In the present study, we aimed to delineate key transcriptomic changes within brain, blood, and HPA/HPT axes tissues in depression by exploring the origin of identified transcriptomic associations, in terms of which genes are likely causal and which genes determine both transcriptomic and phenotypic changes (i.e., pleiotropy). Moreover, we examine how such genes lead the dysregulation of nearby coexpressed genes and of biological pathways. This study will add to the field by aiding the understanding of the biological mechanisms of depression, incorporating several disorder-relevant tissues, and highlighting key genes for the disorder that might be useful candidates for future research on its etiology and treatment.

## Methods and Materials

### Datasets

The analyses used 1) genome-wide summary statistics from the GWAS of MD by Wray *et al.* (5), 2) 21 SNP weight sets from 5 separate transcriptomic reference samples, and 3) the 1000 Genomes Project reference for LD estimation.

First, we leveraged summary statistics from the Psychiatric Genomics Consortium (PGC) MD GWAS (5), which includes information on the genetic susceptibility to MD for 1,185,038 HapMap3 SNPs from 7 samples: the PGC studies, deCODE, Generation Scotland, GERA (Genetic Epidemiology Research on Adult Health and Aging), iPSYCH, UK Biobank, and 23andMe (not publicly accessible). Participants (*n* = 135,458 cases, *n* = 344,901 controls) were of European genetic ancestry.

Second, SNP weights from distinct tissues and samples (of European genetic ancestry) were used. SNP weights represent the correlations of SNPs with the expression of their annotated gene (8). SNP weights from postmortem brain tissue; whole blood; peripheral blood; and adrenal, pituitary, and thyroid glands were downloaded via the TWAS FUSION website (http://gusevlab.org/projects/fusion/#reference-functional-data) after selection based on previous literature (Supplement 1). The weights pertained to the following 5 RNA reference samples: NTR (Netherlands Twin Register) and YFS (Young Finns Study), both of which provide information on blood tissue gene expression; CMC (CommonMind Consortium) and PsychENCODE Consortium, both of which assessed the DLPFC; and the GTEx Consortium, a state-of-the-art study in which expression in multiple brain and peripheral tissues was measured (8,16, 17, 18), although in a limited number of individuals (determining fewer heritable genes). The SNP weights obtained from a given sample and tissue (e.g., GTEx thyroid, NTR peripheral blood) are called SNP weight sets. Each gene within a given SNP weight set is a feature, i.e., a gene that was examined within a given tissue and sample (e.g., *NEGR1* GTEx thyroid)*.* SNP weight set characteristics are presented in Table S1 in Supplement 2.

Third, the 1000 Genomes Phase 3 European LD reference (*N* = 489) (19) was downloaded from the FUSION website (http://gusevlab.org/projects/fusion/).

### Statistical Analyses

All statistical analyses were performed in Bash (GNU Project Bourne Again SHell) or R, version 3.5.0 (The R Project for Statistical Computing, Vienna, Austria). Codes and outputs are publicly available at https://opain.github.io/MDD-TWAS.

#### Transcriptome-wide Significance Threshold

To compute the transcriptome-wide significance threshold for this study, we leveraged a permutation procedure used in a previous TWAS (Supplement 1) (19). This approach estimates a significance threshold adjusted for the number of tested features, accounting for the correlation between features within and across SNP weight sets. The threshold for transcriptome-wide significance was *p* = 1.37 × 10^−6^ (for a false-positive rate of α = .05), with a more stringent significance threshold of *p* = 3.69 × 10^−8^, for α = .001.

#### TWAS FUSION and Colocalization

A TWAS FUSION analysis was run on autosomal chromosomes, following the TWAS FUSION protocol with default settings (Supplement 1) (http://gusevlab.org/projects/fusion/). Colocalization was assessed using the coloc R package for all genes meeting transcriptome-wide significance and within a 1.5-Mb window. This Bayesian approach estimates the posterior probability (PP) that associations within a locus for two outcomes are driven by a shared causal variant. It thus enables the distinction between associations driven by horizontal pleiotropy (1 causal SNP affecting both transcription and MD; posterior probability PP4) and linkage (2 causal SNPs in LD affecting transcription and MD separately; posterior probability PP3). More details are available in Supplement 1.

#### Conditional Analysis

A conditional analysis was used to determine whether multiple significant features within a given locus represent independent associations or a single association owing to correlated predicted expression between features. This was performed using the FUSION software, which jointly estimates the effect of all significant features within each locus by using residual SNP associations with depression after accounting for the predicted expression of other features. This process identifies which features represent independent associations (termed jointly significant) and which features are not significant when accounting for the predicted expression of other features in the region (termed marginally significant) (Supplement 1) (8). We additionally calculated to what extent the GWAS associations within each locus could be explained by functional associations detected in this TWAS (i.e., the variance explained) (Supplement 1).

#### TWAS Fine Mapping

FOCUS, a TWAS association fine mapping method, was used to identify which features are likely to be causal within regions of association (12). Analogous to statistical fine mapping of GWAS results, FOCUS estimates the posterior inclusion probability (PIP) of each feature being causal within a region of association, using the sum of PPs to define the default 90% credible set, a set of features likely to contain the causal feature. The method includes a null model where the causal feature is not present. The PIP of individual features is also of interest, with values > .5 indicating that a feature is more likely to be causal than any other feature in the region. The FOCUS fine mapping function was applied across all SNP weight panels simultaneously without the tissue prioritization option.

#### TWAS-Based Gene Set Enrichment Analysis

Finally, we used a previously applied approach for TWAS-based gene set enrichment analysis (TWAS-GSEA) (19) to identify functionally informed dysregulated pathways characterizing MD (Supplement 1). A linear mixed model was used to test for an association between *z* scores indicating nonzero association for each feature and gene set membership, while adjusting for gene length and numbers of SNPs within the gene region and accounting for correlation between features. Linear mixed models were fitted using the R package lme4qtl (20). For the TWAS-GSEA, we used 7246 hypothesis-free gene sets from MSigDB v6.1 (https://www.gsea-msigdb.org/gsea/msigdb/index.jsp) and 76 candidate gene sets from the GWAS by Wray *et al.* (5). A minimum of 5 genes within the gene set was required to perform the analysis. TWAS-GSEA was run using different sets of TWAS results to identify gene sets enriched across all tissues, within tissue group (brain, blood, HPA/HPT axes), and within each SNP weight set. If multiple features for a single gene were present, the feature explaining the largest amount of variance in the expression of the gene was retained. Multiple testing correction was applied (false discovery rate *q* = .05). Moreover, by using BRAINSPAN data, we investigated the preferential expression of genes at multiple developmental stages (19 stages) (21). This was also done within the mixed model method for TWAS-GSEA, in line with previous literature (19). A Bonferroni significance threshold was used (*p* < .002).

#### Comparisons With Previous Literature

Lastly, we compared our findings with previous literature of observed and predicted gene expression. The comparison was performed to evaluate how the findings from the largest study of observed expression in depression to date (*N* = 1848) (22) compared with ours. This assessed whole-blood gene expression using microarray technology in the Netherlands Study of Depression and Anxiety. Genes were considered as confirmed if their *p* value surpassed a Bonferroni-corrected significance threshold accounting for the number of genes compared across studies (*p* < .05/number of unique genes of nominal significance) in our study. A consistent direction of effect was not a requirement for confirmation of findings. We additionally contrasted our results to 3 previous studies (5,9,10) of predicted gene expression in MD to evaluate the novel contribution of this study. Overall, we considered 136 unique genes, significant in any SNP weight set, in either of the TWASs.

## Results

### TWAS Analysis

**Figure 1:**
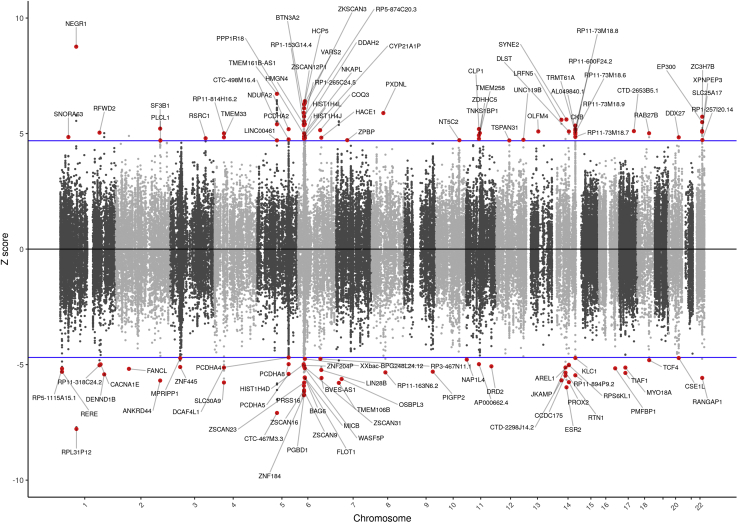
The relationship between gene expression and major depression. Manhattan-style plot of *z* scores for each of the tested genes, across all autosomes and tested single nucleotide polymorphism weight sets. Blue lines indicate the transcriptome-wide significance threshold. The names of statistically significant genes are shown.

We identified 176 significant features, from 94 unique genes, which were differentially expressed (*p* < 1.37 × 10^−6^) across multiple SNP weight sets (i.e., tissues measured within a sample) in MD (Figure 1; Figure S1A, B in Supplement 1; Table S2 in Supplement 2). Of the 176 significant features, 94 were upregulated, while 82 were downregulated. The most significant feature was *NEGR1* (GTEx whole blood) (*z* = 8.76, *p* = 1.94 × 10^−18^). Compared with the GWAS by Wray *et al.* (5), 48 unique genes were novel (based on >500 kb distance and *R*^2^ < .1) (Table S2 in Supplement 2).

The largest number of associations were from the PsychENCODE DLPFC set (22 associated features), but inferences on tissue enrichment are difficult, as SNP weight sets differ in their characteristics (e.g., sample size). Associations are nevertheless shown by tissue group (brain, blood, HPA/HPT axes) in Figure S2A–D in Supplement 1. Of note, 55 associations (from 26 unique genes) were within the extended major histocompatibility complex region (chromosome 6: 26–34 Mb). This region is gene rich and characterized by extensive LD, so these associations should be interpreted with caution. Information on the results once the major histocompatibility complex region is excluded is presented in Supplement 1.

### Colocalization

We evaluated the colocalization status of a gene by calculating the PP that the genetic and functional associations derived from distinct causal SNPs (PP3) and a shared causal SNP (PP4) (23). Of the 176 significant features, 97 (53 unique genes) were considered as colocalized based on their high PP4 (> .8), in line with previous literature (24,25) (Table S3 in Supplement 2). This means that the same genetic variants were driving associations with depression and with these 97 features, potentially suggesting transcriptomic mediation of genetic risk for depression.

### Conditional Analysis

We observed that multiple significant features resided within the same locus (defined as a 1.5 Mb ± 0.5 window), for a total of 36 genomic regions (Table 1; Table S4 in Supplement 2). Conditional analysis of the 176 significant features identified 50 jointly (49 unique genes) and 126 marginally (45 unique genes) significant features. This indicated that most of the identified features were associated with depression owing to their coexpression with the 50 independent features. Importantly, anomalous results were shown at 3 loci (including major histocompatibility complex loci), likely reflecting technical problems owing to the mismatch between the LD references used in the different samples (GWAS and functional data) (Table S4 in Supplement 2).

**Table 1:** Conditional Analysis Findings: Jointly and Marginally Significant Features per Locus

Location	Jointly Significant Features (SNP Weight Set)	No. of Marginally Significant Features	Top TWAS *p*	Top GWAS *p*	Variance Explained, %^a^
chr1:7413452-9875347	*RERE* (YFS blood)	3	1.10 × 10^−7^	3.18 × 10^−8^	100.00
chr1:35885799-37876701	*SNORA63* (GTEx nucleus accumbens)	0	1.24 × 10^−6^	6.27 × 10^−8^	69.30
chr1:71753372-73766162	*NEGR1* (GTEx whole blood)	5	1.94 × 10^−18^	4.54 × 10^−15^	97.80
chr1:174891875-177103690	*RFWD2* (CMC DLPFC splicing)	3	4.66 × 10^−7^	2.30 × 10^−7^	90.00
chr1:180725304-182724241	*CACNA1E* (CMC DLPFC splicing)	0	6.06 × 10^−7^	1.08 × 10^−7^	64.00
chr1:196478918-198741422	*DENND1B* (CMC DLPFC splicing)	2	5.90 × 10^−8^	3.11 × 10^−8^	92.60
chr2:57388379-59467945	*FANCL* (CMC DLPFC splicing, CMC DLPFC)	0	2.18 × 10^−7^	4.68 × 10^−9^	85.00
chr2:196832647-199295649	*ANKRD44* (YFS blood)	1	1.27 × 10^−8^	3.52 × 10^−7^	82.80
chr3:43487406-45561063	*ZNF445* (CMC DLPFC)	0	3.34 × 10^−7^	6.34 × 10^−8^	74.70
chr4:40937584-43090938	*SLC30A9* (GTEx cortex), *TMEM33* (PsychENCODE DLPFC)	8	7.72 × 10^−9^	3.59 × 10^−9^	92.30
chr5:139030460-141219083	*PCDHA5* (GTEx thyroid)	2	6.55 × 10^−8^	1.37 × 10^−6^	87.70
chr6:98832858-100829135	*COQ3* (CMC DLPFC splicing)	0	2.65 × 10^−8^	9.09 × 10^−8^	35.10
chr6:104405706-106583999	*BVES-AS1* (GTEx amygdala)	2	2.43 × 10^−8^	9.50 × 10^−8^	92.90
chr7:11252396-13282905	*TMEM106B* (PsychENCODE DLPFC)	3	7.01 × 10^−9^	2.55 × 10^−8^	100.00
chr7:24021857-26019767	*OSBPL3* (GTEx pituitary)	0	1.88 × 10^−8^	6.49 × 10^−7^	77.70
chr8:51238261-53720740	*PXDNL* (CMC DLPFC)	0	3.92 × 10^−9^	1.34 × 10^−7^	83.80
chr8:60435234-62428932	*RP11-163N6.2* (GTEx thyroid)	0	9.47 × 10^−8^	5.25 × 10^−7^	89.80
chr9:125606617-127604411	*PIGFP2* (PsychENCODE DLPFC)	0	1.12 × 10^−7^	2.73 × 10^−8^	63.80
chr11:56092913-58422547	*TNKS1BP1* (GTEx adrenal gland), *CLP1* (GTEx whole blood)	1	2.04 × 10^−7^	1.47 × 10^−7^	95.20
chr11:60540194-62557903	*TMEM258* (PsychENCODE DLPFC)	0	5.12 × 10^−7^	4.26 × 10^−7^	83.90
chr11:112346414-114345882	*DRD2* (GTEx frontal cortex)	0	3.90 × 10^−7^	4.90 × 10^−7^	0.41
chr13:52652520-54625616	*OLFM4* (CMC DLPFC)	0	3.56 × 10^−7^	6.06 × 10^−19^	29.90
chr14:41077086-43073683	*CTD-2298J14.2* (GTEx thyroid)	2	1.36 × 10^−8^	2.57 × 10^−9^	88.10
chr14:58952573-61334943	*CCDC175* (GTEx thyroid)	3	4.28 × 10^−8^	2.18 × 10^−7^	82.30
chr14:63322572-65770213	*ESR2* (GTEx pituitary)	2	2.20 × 10^−9^	7.60 × 10^−10^	80.00
chr14:74120633-76388050	*PROX2* (GTEx thyroid)	5	8.51 × 10^−9^	6.71 × 10^−9^	93.50
chr14:102878783-105180229	*RP11-894P9.2* (GTEx thyroid)	11	4.69 × 10^−8^	3.05 × 10^−9^	84.60
chr16:71147494-73210261	*PMFBP1* (PsychENCODE DLPFC)	0	2.46 × 10^−7^	3.35 × 10^−8^	76.30
chr17:26406423-28478661	*TIAF1* (GTEx adrenal gland)	1	8.27 × 10^−8^	8.51 × 10^−9^	58.50
chr17:64524284-66521332	*CTD-2653B5.1* (PsychENCODE DLPFC)	0	3.30 × 10^−7^	5.39 × 10^−6^	25.80
chr18:51385406-53561919	*RAB27B* (PsychENCODE DLPFC)	1	5.36 × 10^−7^	3.62 × 10^−11^	14.60
chr20:46838019-48853908	*DDX27* (CMC DLPFC)	0	1.32 × 10^−6^	3.54 × 10^−6^	91.00
chr22:40218102-42697216	*ZC3H7B* (GTEx cerebellum)	8	1.01 × 10^−8^	7.56 × 10^−9^	95.50

Chr, chromosome; CMC, CommonMind Consortium; DLPFC, dorsolateral prefrontal cortex; GTEx, Genotype-Tissue Expression; GWAS, genome-wide association study; NTR, Netherlands Twin Register; SNP, single nucleotide polymorphism; TWAS, transcriptome-wide association study; YFS, Young Finns Study.

a Variance in the top GWAS SNP explained by the top TWAS feature within the locus.

We additionally investigated the effect of adjusting for features’ gene expression on associations between SNPs and the trait (i.e., GWAS findings). The variance in the GWAS associations accounted for by gene expression associations at a given locus ranged from 0.41% to 100%, with a median variance explained of 84%. Genome-wide associations at 3 loci, including *TMEM106B* (PsychENCODE DLPFC), were fully explained by our TWAS results (Table 1). This might mean that at these loci, genetic risk for depression is mediated by functional changes.

### Statistical Fine Mapping

FOCUS was performed to calculate the probability estimates of causality (PIP) for each feature. We found 23 features (23 unique genes) with PIP > .5, indicating these are likely causal in their associations with depression (Table 2; Table S5 in Supplement 2). Of these, 11 were supported by the colocalization analysis (Table 2). The highest probability of causality was within *NEGR1* GTEx whole blood (PIP = 1.00).

**Table 2:** Statistical Fine Mapping Findings: Potentially Causal Features

Location	Gene	SNP Weight Set	FOCUS PIP^a^	Colocalization
chr1:8412457-8877702	*RERE*	YFS blood	.50	True
chr1:36884051-36884179	*SNORA63*	GTEx nucleus accumbens	.70	False
chr1:71861623-72748417	*NEGR1*	GTEx whole blood	1.00	True
chr1:181452685-181775921	*CACNA1E*	CMC DLPFC splicing	.52	False
chr1:197473878-197744623	*DENND1B*	CMC DLPFC	.73	True
chr2:58386377-58468515	*FANCL*	CMC DLPFC splicing	.80	True
chr4:41992489-42092474	*SLC30A9*	GTEx cortex	.53	False
chr5:87988462-87989789	*CTC-467M3.3*	GTEx frontal cortex	.84	True
chr6:99817347-99842082	*COQ3*	CMC DLPFC splicing	.97	False
chr6:105584224-105617820	*BVES-AS1*	GTEx amygdala	.74	False
chr7:12250867-12282993	*TMEM106B*	GTEx whole blood	.61	True
chr7:24836158-25021253	*OSBPL3*	GTEx pituitary	.98	False
chr8:52232136-52722005	*PXDNL*	CMC DLPFC	1.00	False
chr8:61297147-61429354	*RP11-163N6.2*	GTEx thyroid	.92	False
chr9:126605315-126605965	*PIGFP2*	PsychENCODE DLPFC	.94	False
chr11:113280318-113346111	*DRD2*	GTEx frontal cortex	.97	False
chr13:53602875-53626196	*OLFM4*	CMC DLPFC	.99	False
chr14:42076773-42373752	*LRFN5*	GTEx cerebellum	.50	True
chr14:59971779-60043549	*CCDC175*	GTEx thyroid	.61	True
chr14:64550950-64770377	*ESR2*	GTEx pituitary	.59	True
chr14:75319736-75330537	*PROX2*	GTEx thyroid	.72	True
chr16:72146056-72210777	*PMFBP1*	PsychENCODE DLPFC	.96	False
chr17:27401933-27405875	*TIAF1*	GTEx adrenal gland	.75	True

Chr, chromosome; CMC, CommonMind Consortium; DLPFC, dorsolateral prefrontal cortex; GTEx, Genotype-Tissue Expression; PIP, posterior inclusion probability; SNP, single nucleotide polymorphism; YFS, Young Finns Study.

a PIP estimate of causality.

### High-Confidence Associations

In a data-driven approach, we highlighted the genes that are most relevant to MD (high-confidence associations). First, in the TWAS results, we applied a more stringent significance threshold of *p* < 3.69 × 10^−8^ (α = .001) to minimize the chance of false-positive results (26). Second, from these highly significant genes, we identified the genes that were colocalized (PP4 > .8) and likely to be causal (PIP > .5). These features were therefore strongly associated with MD, possibly at a causal level, and their transcription changes resulted from the corresponding genetic risk for depression. This strategy showed 6 high-confidence associations (Table 3), from brain tissues (frontal cortex, cerebellum) and peripheral tissues (whole blood, thyroid, pituitary). The *NEGR1* gene (GTEx whole blood) constituted the most significant hit (*p* = 1.94 × 10^−18^) with the highest probability of causality (PIP = 1.00).

**Table 3:** Characteristics of High-Confidence Associations: Highly Significant, Colocalized, and Potentially Causal Features

Location	Gene	SNP Weight Set	TWAS *z*	TWAS *p*	Novel^a^	Colocalization PP4^b^	FOCUS PIP^c^
chr1:71861623-72748417	*NEGR1*	GTEx whole blood	8.76	1.94 × 10^−18^	No	.99	1.00
chr5:87988462-87989789	*CTC-467M3.3*	GTEx frontal cortex	−7.09	1.33 × 10^−12^	No	.85	.84
chr7:12250867-12282993	*TMEM106B*	GTEx whole blood	5.53	3.18 × 10^−8^	Yes (GWAS)	.99	.61
chr14:42076773-42373752	*LRFN5*	GTEx cerebellum	5.60	2.17 × 10^−8^	No	.96	.50
chr14:64550950-64770377	*ESR2*	GTEx pituitary	−5.98	2.20 × 10^−9^	No	.86	.59
chr14:75319736-75330537	*PROX2*	GTEx thyroid	−5.76	8.51 × 10^−9^	Yes (TWAS)	.96	.72

Chr, chromosome; GTEx, Genotype-tissue expression; GWAS, genome-wide association study; PIP, posterior inclusion probability; PP, posterior probability; SNP, single nucleotide polymorphism; TWAS, transcriptome-wide association study.

a Genes are novel compared with GWAS if >500 kb away from a lead GWAS variant and if their predicted expression is not correlated with GWAS variant (*R*^2^ < .1). Genes are novel compared with previous TWASs of major depression if these did not identify them as statistically significant.

b PP of colocalization.

c PIP estimate of causality.

### TWAS-Based Gene Set Enrichment Analysis

Candidate gene analysis highlighted enrichment for 15 gene sets, including neuronal and synaptic processes, schizophrenia genetic risk, and *RBFOX2*, a key regulator of splicing within the nervous system and of estrogen receptor transcriptional activity (Table 4) (27). The hypothesis-free analysis revealed enrichment for 7 gene sets, including macromolecular and protein complex binding (Table 5). Enrichment or depletion of our differentially expressed genes was not found at any developmental stage (Figure S3A–C in Supplement 1).

**Table 4:** Candidate Gene Set Enrichment Results Based on TWAS Results From All Tissues, Tissue Sets, and Each Panel

Gene Set	PubMed ID	Estimate	SE	*t*	*p*	FDR *p*^a^	Tissue
All Tissue Results							
RBFOX2	24613350	0.08	0.02	3.26	5.57 × 10^−4^	3.95 × 10^−2^	All
Tissue-Set Results							
RBFOX2	24613350	0.11	0.03	4.13	1.78 × 10^−5^	1.26 × 10^−3^	Brain
SCZ.COMPOSITE	24463508	0.11	0.03	3.64	1.39 × 10^−4^	4.92 × 10^−3^	Brain
RBFOX1.RBFOX3	24613350	0.08	0.02	3.45	2.80 × 10^−4^	6.62 × 10^−3^	Brain
FMRP	21784246	0.12	0.04	3.35	3.98 × 10^−4^	7.06 × 10^−3^	Brain
POTENTIALLY.SYNAPTIC.ALL	27694994	0.06	0.02	3.03	1.21 × 10^−3^	1.72 × 10^−2^	Brain
PGC.BP.P10.4	21926972	0.18	0.06	2.85	2.20 × 10^−3^	2.60 × 10^−2^	Brain
NEURONAL.PSD	23071613	0.09	0.03	2.67	3.83 × 10^−3^	3.89 × 10^−2^	Brain
Tissue-Specific Results							
MIR.137	24463508	0.36	0.10	3.58	1.70 × 10^−4^	1.02 × 10^−2^	CMC DLPFC RNA-seq
SCZ.DENOVO.NONSYN	24463508	0.40	0.12	3.51	2.25 × 10^−4^	1.17 × 10^−2^	GTEx pituitary
SCZ.COMPOSITE	24463508	0.23	0.07	3.28	5.21 × 10^−4^	1.36 × 10^−2^	GTEx pituitary
SCZ.COMPOSITE	24463508	0.27	0.08	3.19	7.03 × 10^−4^	3.24 × 10^−2^	GTEx basal ganglia
CONSTRAINED	25086666	0.27	0.09	2.97	1.50 × 10^−3^	3.44 × 10^−2^	CMC DLPFC RNA-seq
RBFOX1.RBFOX3	24613350	0.12	0.04	2.93	1.72 × 10^−3^	3.44 × 10^−2^	CMC DLPFC RNA-seq
PGC.SCZ.P10.4	24463508	0.27	0.10	2.73	3.20 × 10^−3^	4.79 × 10^−2^	CMC DLPFC RNA-seq

CMC, CommonMind Consortium; DLPFC, dorsolateral prefrontal cortex; FDR, false discovery rate; GTEx, Genotype Tissue Expression; RNA-seq, RNA sequencing; SE, standard error; TWAS, transcriptome-wide association study.

a Multiple testing–corrected *p* value using FDR method.

**Table 5:** Hypothesis-free Gene Set Enrichment Results Based on TWAS Results From Each Panel

Gene Set	Estimate	SE	*t*	*p*	FDR *p*^a^	Tissue
GO.MACROMOLECULAR.COMPLEX.BINDING	0.46	0.09	5.07	1.98 × 10^−7^	5.99 × 10^−4^	GTEx basal ganglia
GO.MICROTUBULE.BINDING	1.04	0.22	4.68	1.46 × 10^−6^	2.20 × 10^−3^	GTEx basal ganglia
GO.ALCOHOL.BINDING	1.79	0.38	4.66	1.56 × 10^−6^	5.16 × 10^−3^	GTEx pituitary
GO.CHROMATIN.BINDING	0.79	0.19	4.19	1.42 × 10^−5^	1.41 × 10^−2^	GTEx basal ganglia
GO.PROTEIN.COMPLEX.BINDING	0.44	0.11	4.12	1.87 × 10^−5^	1.41 × 10^−2^	GTEx basal ganglia
GO.LIGAND.DEPENDENT.NUCLEAR.RECEPTOR.BINDING	1.98	0.50	3.98	3.47 × 10^−5^	2.09 × 10^−2^	GTEx basal ganglia
GO.REGULATION.OF.INTRINSIC.APOPTOTIC.SIGNALING.PATHWAY	1.79	0.43	4.14	1.73 × 10^−5^	3.30 × 10^−2^	GTEx amygdala

FDR, false discovery rate; GTEx, Genotype Tissue Expression; SE, standard error; TWAS, transcriptome-wide association study.

a Multiple testing–corrected *p* value using FDR method.

### Comparison With Previous Literature

First, our findings were compared with the largest study of observed gene expression to date for MD (22). Of the significant genes (false discovery rate < 0.1) of such study, we also identified 6 unique genes (*PAPPA2, MBNL1, TMEM64, GNPTAB, KTN1, TMED10*). Four of these (*PAPPA2*, *GNPTAB, KTN1, TMED10*) were consistently upregulated or downregulated (Table S6 in Supplement 1; Supplement 1).

To evaluate the novelty of our findings, we compared our results with previous studies of predicted gene expression changes in MD performed by Wray *et al.* (5) (TWAS FUSION method), Gaspar *et al.* (9) (S-PrediXcan), and Gerring *et al.* (10) (S-PrediXcan). Our results were overlapping with previous TWASs at 83 genes (Table S7 in Supplement 2). Moreover, across all TWASs performed to date, we identified 53 novel associations (unique genes). This might be due to differences in the type and number of SNP weight sets used, methods, and statistical thresholds (e.g., permutation-based vs. Bonferroni).

## Discussion

This is the first study to uncover the genetic component of gene expression in MD through a comprehensive investigation in multiple tissues and an in-depth characterization of the identified associations delineating key transcriptomic changes. Here, we highlight a few key findings. First, we detected 94 unique genes associated with depression, approximately half of which were novel. Second, with a series of follow-up analyses, we found 6 associations that were of high confidence, based on their strong TWAS associations, colocalization, and probability of causality. These findings highlight the transcriptomic changes in MD that likely play a role in etiology of the disorder, resulting from the same genetic variations as the disorder and determining, in turn, transcriptomic changes at nearby genes and pathways involved in key biological processes. Such findings on key genes for depression can guide future research on drug targets as well as candidate gene investigations in animal studies, where consequences of molecular alterations can be more readily observed by inducing gene knockdown or upregulation.

### Key Findings

When testing for an association between gene expression and MD, we detected 94 transcriptome-wide significant genes, differentially expressed across multiple tissues, thus demonstrating the presence of widespread transcriptomic changes in depression. Comparison with previous literature highlighted the novelty of this study, which enabled the identification of 48 (compared with GWAS findings) and 53 (compared with previous TWASs) novel genes.

Further investigation of significant associations through a conditional analysis determined whether gene associations within the same genomic region represent independent associations or whether multiple genes are associated owing to correlated predicted expression. The 94 significant genes represented 49 independent associations with depression, suggesting that approximately half of the identified signal depends on LD and correlated predicted expression of nearby genes. The strength of association for each feature can be affected by different characteristics of the SNP weight sets (e.g., sample size), thus warranting cautionary interpretations of which features are driving the associated loci. Comparison of the GWAS summary statistics before and after conditioning on significant TWAS associations additionally revealed that GWAS associations were explained to a major extent by TWAS associations, further suggesting the possibility of transcriptomic mediation of genetic risk for depression.

When exploring whether significant associations were driven by pleiotropy or linkage using a colocalization analysis, we observed that 53 transcription-MD relationships derived from the same causal polymorphisms underlying SNP-MD associations. This indicated that most of the detected genes constituted pleiotropic effects as opposed to linkage. While these findings suggest that transcription mediates the relationship between genetic susceptibility and depression, colocalization does not test for such relationships, and it cannot identify specific causal variants (8).

To gain further insight into which genes are likely causal for MD, we used a TWAS fine mapping approach called FOCUS. In some instances, FOCUS clearly highlighted a single feature as the causal association, such as the upregulation of *NEGR1* in the blood. Of the 23 genes with a high probability of causality estimated by FOCUS, 11 were identified as colocalized. A key distinction between these methods is that colocalization assumes a single causal variant, whereas FOCUS allows for multiple causal variants.

Six high-confidence associations were identified. Notably, none of these were found by previous TWASs on other psychiatric phenotypes (e.g., bipolar disorder) (11,28,29). Of the high-confidence associations, 3 should be highlighted owing to their functional role: *NEGR1*, *ESR2*, and *TMEM106B*. SNPs within these 3 genes have been previously detected in one or more GWASs of depression (4,5,30). Our study contributed to previous literature by elucidating the functional characteristics of such genes, showing upregulation for *NEGR1* and *TMEM106B* and downregulation for *ESR2.*

*NEGR1* plays a role in axon extension, synaptic plasticity, and synapse formation, processes key to neuronal functioning (31, 32, 33). Moreover, it includes variants found to be related to obesity, a trait repeatedly correlated with MD, at both phenotypic and biological levels (34). *ESR2* regulates the activity of estrogen, a sex hormone involved in HPA axis activity and inflammation (35,36). Estrogen fluctuations across a woman’s life span, for example, during premenstrual monthly period and menopause, have been proposed as a risk factor for depression (37). However, it remains unclear whether the risk is driven by estrogen or other co-occurring hormonal changes (37,38). *TMEM106B* was previously implicated as a susceptibility gene for neurodegenerative disorders (39, 40, 41) and TDP-43 abnormalities (40), which are featured in such pathologies (40, 41, 42). Depression has been repeatedly suggested as a risk factor for neurodegenerative disorders (43, 44, 45). Moreover, TDP-43 proteinopathy was shown in a small sample of patients with late-life depression (46). Overall, while previous literature points to an important role of these genes in depression and related phenotypes, replication of associations is necessary as well as greater insight into the relationship between estrogen levels and TDP-43 with MD.

TWAS-GSEA was able to identify several gene sets showing dysregulated expression in individuals with MD. The enriched gene sets are congruent with a previous GWAS-based enrichment analysis (5), corroborating the importance, among others, of genes bound by transcription factors (RBFOX1, RBFOX2, RBFOX3, FMRP) and genes encoding synaptic proteins and ion channels. Novel enriched pathways were also found for key biological functions such as protein and macromolecular complex binding. Replication is warranted.

### Limitations of Present Study and Suggestions for Future Research

While these findings are promising, several limitations merit discussion. First, the small sample sizes of the gene expression reference samples may have impeded the detection of subtle effects of the transcriptome on depression, meaning that larger samples are needed. Second, the wide range of MD definitions within the GWAS samples, ranging from self-reported depression (e.g., 23andMe) to clinical diagnosis (e.g., iPSYCH), might impact results. Future studies could investigate to what extent minimal phenotyping affects findings at the transcriptomic level compared with more robust definitions. Third, we analyzed a wider set of tissues than previous MD TWASs, with 21 distinct SNP weight sets from blood, brain, and HPA/HPT axes. This may uncover more true associations but may also have introduced noise, as using tissues not strictly relevant to depression might capture noncausal genes (47). Nonetheless, tested tissues were selected based on previous literature, meaning that such tissues are supposedly disease relevant. An alternative strategy would be to use data-driven tissue selection, leveraging an LD score regression-based method (48). Moreover, valuable eQTL data (e.g., eQTLGen Consortium) were not used because of the lack of precomputed SNP weight and access to individual-level data (for weights development). We nevertheless recommend the calculation of such weights when possible. Furthermore, our TWAS approach, by solely assessing the *cis*-genetic component of gene expression, cannot capture *trans*-eQTL effects. Future research should channel resources toward building larger gene expression reference panels to enable investigation of *trans-*eQTL effects. Lastly, while this study provided further insight into the relationship between SNPs, gene expression, and depression and used colocalization and causal fine mapping analyses to test certain criteria of a causal model, it cannot verify causality between associated genes and depression.

In conclusion, we provide evidence for widespread transcriptomic changes in MD. Our study enables the detection of novel associations and the elucidation of the transcriptomic changes that previously identified risk genes undergo. We underline genes that might be of key relevance to depression, including *NEGR1, ESR2,* and *TMEM106B*. These results suggest an important role of the genetic component of gene expression in depression.

## Supplementary Material

Supplement 2

Key Resources Table
