# Supplementary material for: Delineating the Genetic Component of Gene Expression in Major Depression
Source: Biol Psychiatry. 2021 Mar 15;89(6):627–36. doi: 10.1016/j.biopsych.2020.09.010 (PMC7886308; doi:10.1016/j.biopsych.2020.09.010)
Supplement: Supplement 1 [file mmc1.pdf]

Delineating the Genetic Component of Gene Expression in Major Depression

Supplement 1

Supplementary Text

Datasets

The Major Depression GWAS summary statistics were obtained from the Psychiatric Genomics Consortium (https://www.med.unc.edu/pgc/). For TWAS analysis using the FUSION-released SNP-weights, the GWAS summary statistics were converted to LD-score format using the LDSC munge_stats.py utility using Python (version 3.5.1). The LDSC munge_stats.py software restricts variants to HapMap3 variant. However, PsychENCODE SNP-weights are not restricted to HapMap3 variants. Therefore, for analyses including the PsychENCODE SNP-weights (PsychENCODE TWAS, conditional analysis, FOCUS analysis), the GWAS summary statistics were formatted using the FOCUS munge function, as this avoids restricting the GWAS summary statistics to HapMap3 variants.

SNP-weight sets from peripheral tissues available in the TWAS FUSION website were selected based on a comprehensive literature search. SNP-weight sets were chosen if they were derived from tissues previously found as molecularly dysregulated in depression (e.g. transcriptomically) in more than one study. Previous literature was explored through the "Google Scholar" search engine based on a combination of the tissue name and of the following terms: "depression review", "MD review", "gene expression in depression", and "depression". SNP-weights from *postmortem* brain tissue, whole blood, peripheral blood, and the adrenal, pituitary, and thyroid glands were finally downloaded from the TWAS FUSION website

(http://gusevlab.org/projects/fusion/#reference-functional-data). Brain tissues were selected due to their disorder relevance. Literature additionally showed that molecular changes in depression were present in peripheral/whole blood tissues (1–7), adrenal gland (8–11), pituitary gland (12–15), and thyroid (16–20).

SNP-weights were derived from several studies, including the PsychENCODE cohort. The PsychENCODE SNP-weights were downloaded from the PsychENCODE resources page (http://resource.psychencode.org/) section titled 'Cross-Disorder Analysis TWAS weights'. The downloaded SNP-weights were in FUSION format although some differences to FUSION-released SNP-weights existed. The variant IDs within the PsychENCODE SNP-weights were chromosome and bp position (CHR:BP), instead of RSIDs as is used in FUSION released SNP-weights. Using the 1KG Phase 3 reference, the variant IDs were updated to RSIDs to enable combined analysis of PsychENCODE SNP-weights with SNP-weights released by FUSION. Furthermore, PsychENCODE SNP-weights were based on all variants available in the PsychENCODE cohort after HRC imputation, instead of being restricted to HapMap3 variants like FUSION-released SNP-weights. To address this, a 1KG Phase 3 reference that was not restricted to HapMap3 variants was used in the PsychENCODE TWAS analysis, and downstream conditional and FOCUS analyses across PsychENCODE and FUSION-released SNP-weights sets. Most common variants in the HRC reference are available in the 1KG Phase 3 reference, although the few missing variants may lead to a small decrease in gene expression imputation accuracy.

Statistical analyses

Calculation of the transcriptome-wide significance threshold

Firstly, as expected in the FUSION protocol, GE levels were inferred for all heritable features based on the selected SNP-weight sets and the 1000 Genomes LD reference data. GWAS summary

statistics were not used as the calculation of the significance threshold should be based on a null phenotype. A thousand permutations were performed, with each permutation randomly generating a normally-distributed null phenotype. Such phenotype was subsequently tested in association with predicted GE levels. Given the use of a random phenotype, the identified feature – trait relationships should constitute false-positive findings, uniquely attributable to chance. To develop a significance threshold able to distinguish between chance and meaningful findings, the minimum p-value of all individual permutations ($N_{permutations}$ = 1,000) was collected to form a normal distribution of minimum p-values. The five percent quantile of this distribution, equalling to a false positive rate of $\alpha = .05$ (21), was considered as our transcriptome-wide significance threshold, which corresponded to $p = 1.37\times10^{-06}$. We additionally calculated a more stringent threshold to capture genes of high significance ($\alpha = 0.001$): $p = 3.69\times10^{-08}$.

TWAS FUSION

Gene expression-depression associations were obtained only for features with a non-zero *cis*-SNP heritability ($p < 0.01$). This was calculated with the Average Information Restricted Maximum Likelihood (AI-REML) algorithm of Genome-wide Complex Trait Analyses (GCTA). SNPs were selected if they resided within the gene boundaries ±500kb.

A TWAS FUSION analysis was run for all significantly heritable features, for each feature, based on one of several predictive models (BLUP, LASSO, elastic net, or BSLMM), with the best-fitting model being used. TWAS Z-scores were estimated, for each feature separately, based on the following linear model:

$$\text{Feature } Z\text{-score} = w_1z_1 + w_2z_2 + w_3z_3 + w_4z_4 \ldots$$

Where w_i = the correlation of a SNP within a feature with the gene expression of such feature,

z_i = the standardized effect from the GWAS which tested the association between that SNP and a trait, and i = the SNP within the feature.

Outputs from all chromosomes and SNP-weight sets were merged and subsequently filtered based on our transcriptome-wide significant threshold.

Colocalization

This method used a Bayesian approach estimating the posterior probability (PP) of five models concerning GWAS and TWAS associations. These involve a SNP being in:

PP0) No association with depression or GE (null findings),

PP1) Association with depression only (GWAS significance),

PP2) Association with GE only (TWAS significance),

PP3) Association with both, from two independent SNPs (GWAS and TWAS significance, two SNPs involved),

PP4) Association with both, at a shared SNP (GWAS and TWAS significance, one SNP only).

By comparing values for models three and four (PP3 vs. PP4), we can distinguish whether the GWAS and TWAS associations are colocalized, i.e. whether the signal for an association with the trait and with GE at a locus results from the same causal polymorphism. To perform colocalization, the *coloc* R package (22), available in the FUSION software, was employed. Colocalization was implemented only for genes surpassing transcriptome-wide significance.

Conditional Analysis

With a conditional analysis, we could identify loci of co-expression as well as distinguish between independent and conditioned features. *Independent/jointly significant features* remain associated

with the phenotype, at a nominal significance level ($p < 0.05$), after adjustments. *Conditioned* or *marginally significant features* are those whose association with depression is solely reliant on the expression of other nearby features. Conditioned features are thus significantly associated with depression in the *un*adjusted model only.

A conditional analysis can additionally show to what extent GWAS signal is attributable to functional associations. In fact, in such analysis, the top SNP in a locus is conditioned on the GE patterns of the most significant feature in the same locus. Subsequently, the variance in a SNP signal accounted for by such functional associations was calculated, with the following formula:

$$R^2 = 1 - \chi^2_{\textit{conditioned GWAS association}} / \chi^2_{\textit{unconditioned GWAS association}}$$

Where R^2 = variance explained,

$\chi^2_{\textit{conditioned GWAS association}}$ = SNP-phenotype association after adjusting for the GE of the most significant feature in the locus,

$\chi^2_{\textit{unconditioned GWAS association}}$ = SNP-phenotype association before adjusting.

The conditional analysis was performed for chromosomal regions with multiple statistically significant features, within and across SNP-weight sets, as described in the FUSION webpage (http://gusevlab.org/projects/fusion/). Features were defined as pertaining to a shared locus when boundaries overlapped within 1.5Mb±0.5.

TWAS-GSEA

For the TWAS-GSEA, a linear mixed model was run. Gene set membership was regressed on the TWAS feature *z*-score indicating non-zero association. Gene set membership was developed as a dichotomous variable with information on whether a gene pertains to a given functional

annotation. Analyses were performed with the R package lme4qtl (23), which permits the fitting of linear mixed models.

We conducted a *hypothesis-free* TWAS-GSEA testing all annotations from the gene ontology (GO) resource, a database where gene and gene set functions are specified. *Candidate gene sets* were also tested. These contained pathways previously selected by Wray et al. (24) due to their involvement in psychiatric disorders. We attempted to validate these with a functionally-informed gene set enrichment analysis.

Comparison with previous literature

We additionally contrasted our results to previous studies of observed gene expression and of predicted gene expression (TWASs) in MD. The largest study to date on observed gene expression (1) was used for comparison (N = 1,848). This leveraged data from the Netherlands Study of Depression and Anxiety, which is a cohort also included in the Wray et al. GWAS (24).

To date, only three TWASs of MD, with significant findings, have been published: one by Wray et al. (24), one by Gaspar et al. (25), and one by Gerring et al. (26). These studies utilized the same or similar GWAS summary statistics to the ones we employed, but they selected different SNP-weight sets (i.e. Wray et al.: CMC DLPFC only, Gaspar et al.: all GTEx brain tissues and the Depression Genes and Network whole blood weights, Gerring et al.: all GTEx brain and blood tissues). Compared to these studies, we chose a wider range of SNP-weight sets (e.g. PsychENCODE DLPFC, GTEx Pituitary, Thyroid, Adrenal tissues). Of note, while Wray et al. employed a TWAS FUSION approach, Gaspar et al. and Gerring et al. performed an S-PrediXcan analysis.

Results

Results after exclusion of the Major Histocompatibility Complex

Here, we explain results from the TWAS and follow-up analyses after excluding features from the Major Histocompatibility Complex (MHC). This was done as associations in the MHC region are generally difficult to interpret due to extensive linkage disequilibrium.

Firstly, we identified 121 transcriptome-wide significant features. Of these, 77 were colocalized and 40 were jointly significant features. When using FOCUS fine-mapping, 23 features were likely causal. All six of the high-confidence associations were outside the MHC. Moreover, 38 features were novel compared to the Wray et al. GWAS (24).

Supplementary Figures

Supplementary Figure S1A-B

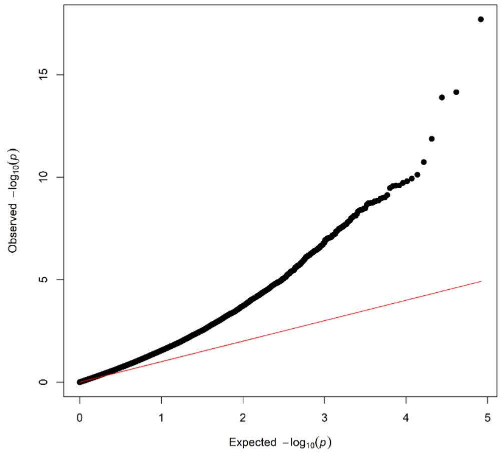

A.

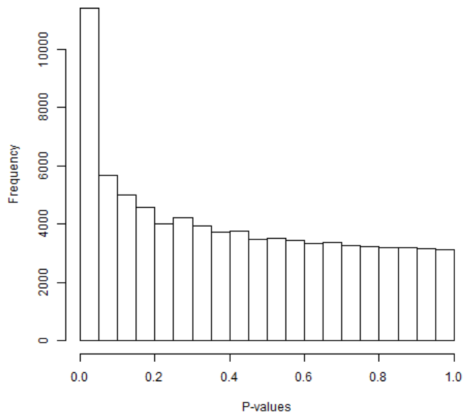

B.

The relationship between major depression and the genetic component of gene expression. (A) QQ-plot showing the distribution of *p*-values expected under the null hypothesis (red line) versus the observed distribution (black line). Inflation is observed. In line with the Wray et al. GWAS results, this is likely the result of polygenicity as opposed to linkage disequilibrium. (B) Histogram of *p*-values: the equal distribution of *p*-values at the bottom represents the null hypothesis being met. The peak in correspondence to the smallest *p*-values provides evidence for our alternative hypothesis of an association between depression and GE.

Supplementary Figure S2A-D

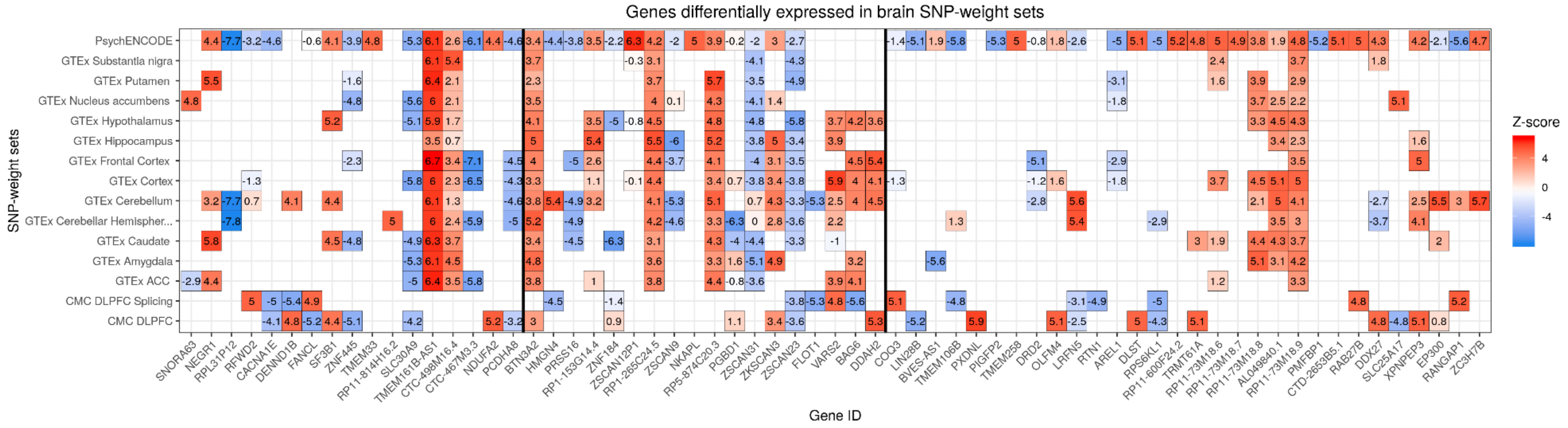

A.

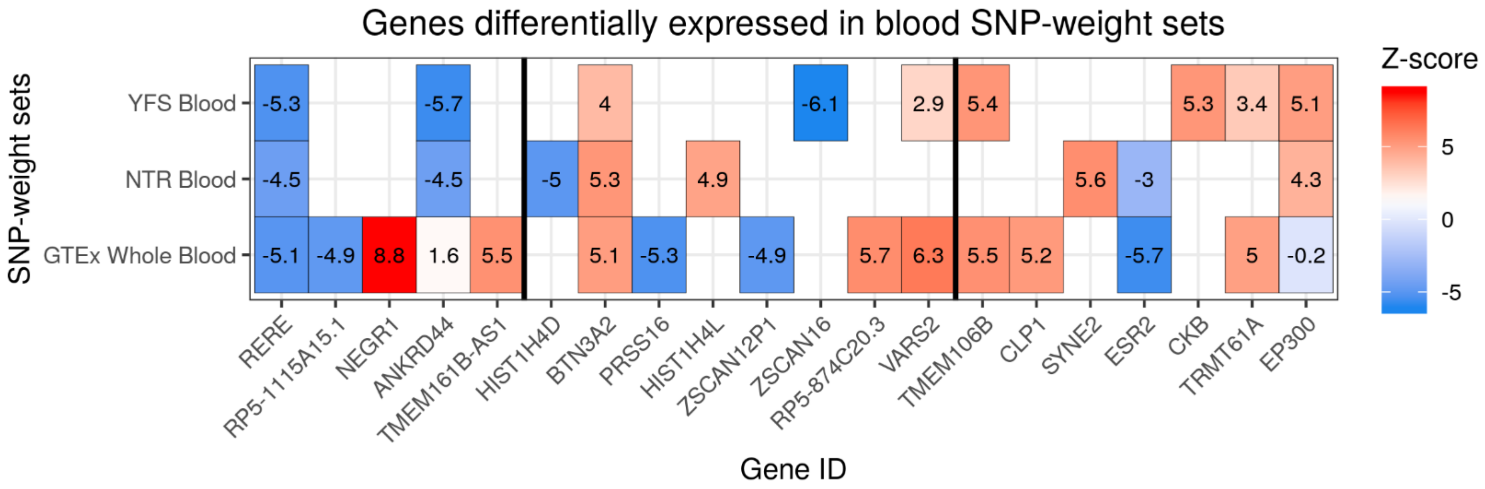

B.

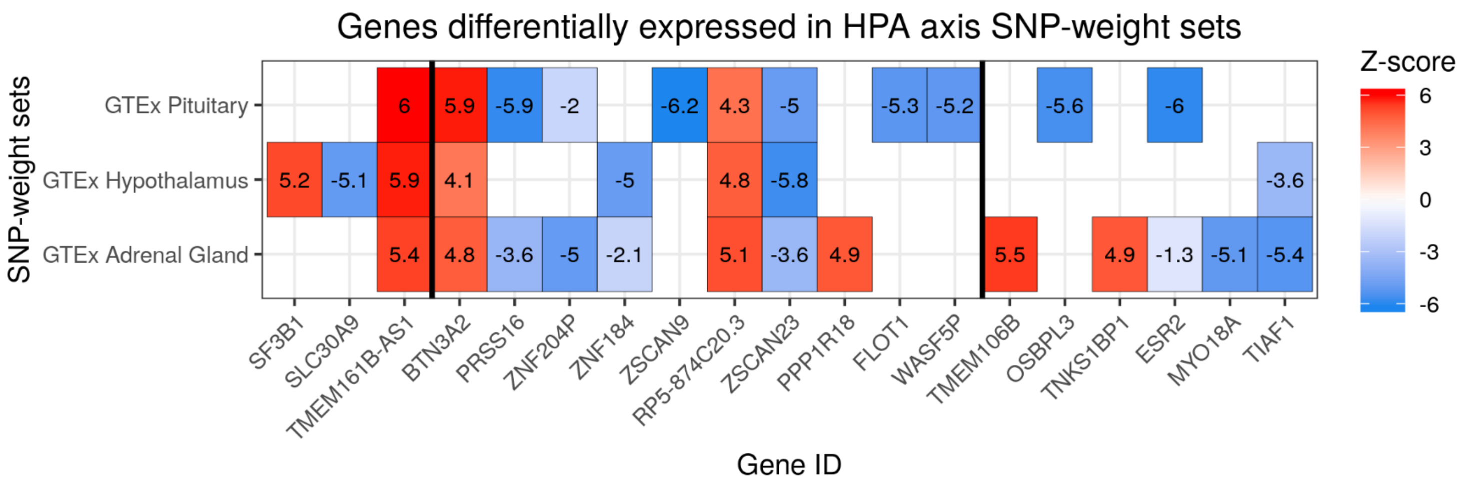

C.

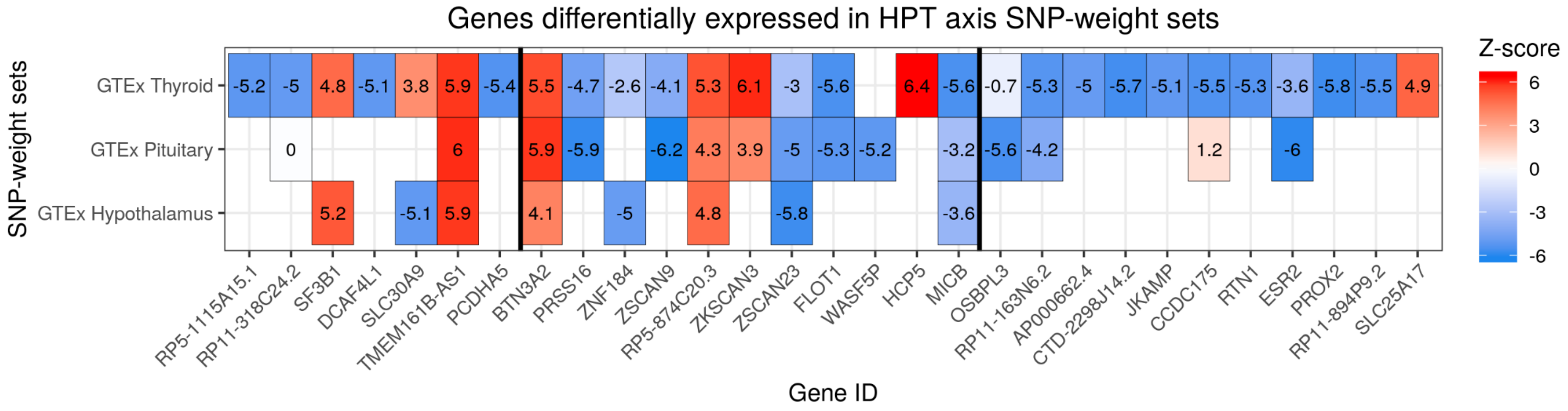

D.

Comparisons of z-scores across SNP-weight sets for heritable genes expressed (A) across brain SNP-weight sets, (B) across blood SNP-weight sets, (C) across HPA axis SNP-weight sets, (D) across HPT axis SNP-weight sets. White spaces corresponded to genes that were

not tested in the TWAS FUSION analysis due to their not significant heritability. Blue shades indicate downregulation while red ones represent upregulation of gene expression in depression. Black vertical lines indicate where the major histocompatibility complex region starts and ends. Z-scores > |4.83| were transcriptome-wide significant.

ACC = Anterior Cingulate Cortex; CMC = CommonMind Consortium; DLPFC = Dorsolateral Prefrontal Cortex; GTEx = Genotype-tissue expression; NTR = Netherlands Twins Register; YFS = Young Finns Study

Supplementary Figure S3A-C

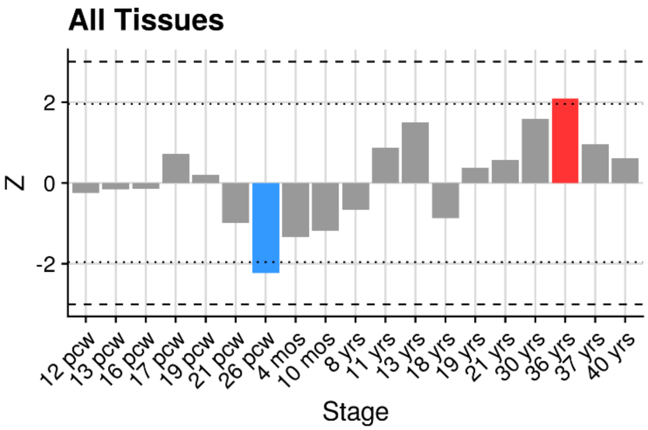

A.

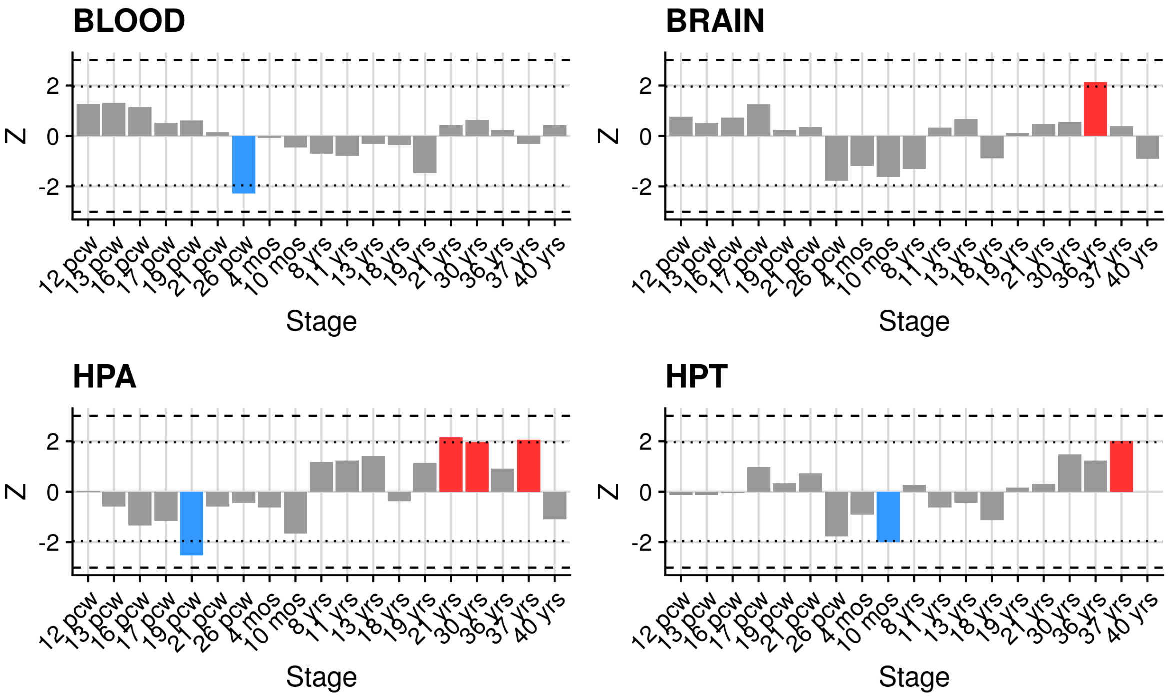

B.

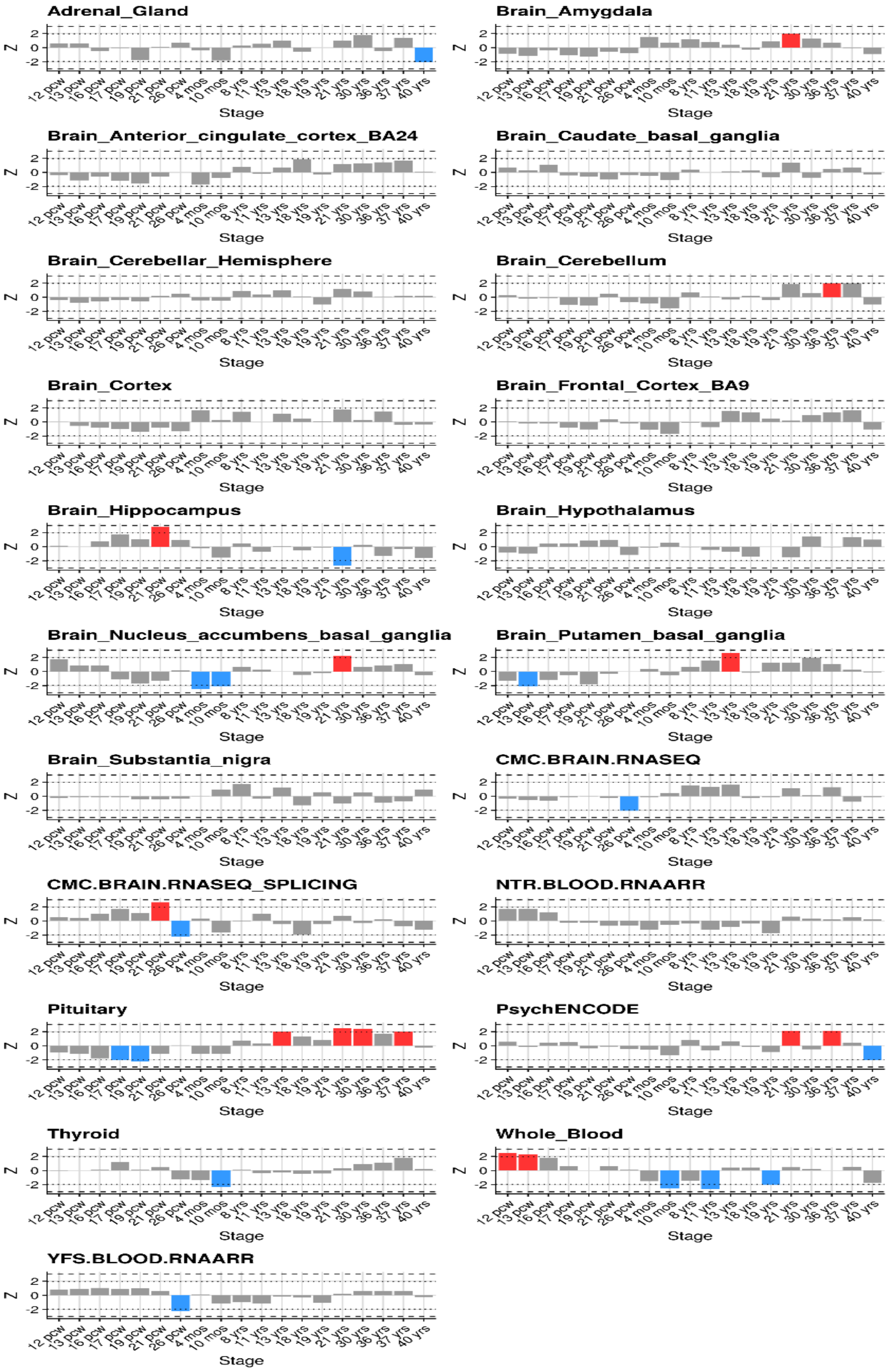
Adrenal_Gland
Brain_Amygdala
Brain_Anterior_cingulate_cortex_BA24
Brain_Caudate_basal_ganglia
Brain_Cerebellar_Hemisphere
Brain_Cerebellum
Brain_Cortex
Brain_Frontal_Cortex_BA9
Brain_Hippocampus
Brain_Hypothalamus
Brain_Nucleus_accumbens_basal_ganglia
Brain_Putamen_basal_ganglia
Brain_Substantia_nigra
CMC.BRAIN.RNASEQ
CMC.BRAIN.RNASEQ_SPLICING
NTR.BLOOD.RNAARR
Pituitary
PsychENCODE
Thyroid
Whole_Blood
YFS.BLOOD.RNAARR
Z
2
0
-2
12 pcw
13 pcw
16 pcw
17 pcw
19 pcw
21 pcw
26 pcw
4 mos
10 mos
8 yrs
11 yrs
13 yrs
18 yrs
19 yrs
21 yrs
30 yrs
36 yrs
37 yrs
40 yrs
Stage

C.

Levels of expression for differentially expressed genes throughout developmental stages and across (A) all tissues, (B) groups of tissues (brain, blood, HPT and HPA axes), and (C) single tissues. No Bonferroni significant enrichment or depletion was shown. Nominally significant enrichment is however depicted in red, while nominally significant depletion is depicted in blue. Enrichment indicates that differentially expressed genes were particularly expressed at a given developmental period. On the contrary, depletion shows that differentially expressed genes were generally expressed at lower rates at a given developmental period.

Supplementary References

1. Jansen R, Penninx BWJH, Madar V, Xia K, Milaneschi Y, Hottenga JJ, *et al.* (2016): Gene expression in major depressive disorder. *Mol Psychiatry* 21: 339–347.

2. Miller AH, Raison CL (2016): The role of inflammation in depression: from evolutionary imperative to modern treatment target. *Nat Rev Immunol* 16: 22–34.

3. Chen D, Meng L, Pei F, Zheng Y, Leng J (2017): A review of DNA methylation in depression. *J Clin Neurosci* 43: 39–46.

4. Mostafavi S, Battle A, Zhu X, Potash JB, Weissman MM, Shi J, *et al.* (2014): Type I interferon signaling genes in recurrent major depression: increased expression detected by whole-blood RNA sequencing. *Mol Psychiatry* 19: 1267–1274.

5. Ciobanu L, Sachdev PS, Trollor JN, Reppermund S, Thalamuthu A, Mather KA, *et al.* (2017): Genome-Wide Gene Expression Signature Of Depression. *Eur Neuropsychopharmacol* 27: S484.

6. Maffioletti E, Cattaneo A, Rosso G, Maina G, Maj C, Gennarelli M, *et al.* (2016): Peripheral whole blood microRNA alterations in major depression and bipolar disorder. *J Affect Disord* 200: 250–258.

7. Zheleznyakova GY, Cao H, Schiöth HB (2016): BDNF DNA methylation changes as a biomarker of psychiatric disorders: literature review and open access database analysis. *Behav Brain Funct* 12: 17.

8. Parker KJ, Schatzberg AF, Lyons DM (2003): Neuroendocrine aspects of hypercortisolism in major depression. *Horm Behav* 43: 60–66.

9. Rubin RT, Phillips JJ, Sadow TF, McCracken JT (1995): Adrenal Gland Volume in Major Depression: Increase During the Depressive Episode and Decrease With Successful Treatment. *Arch Gen Psychiatry* 52: 213–218.

10. Kahl KG, Schweiger U, Pars K, Kunikowska A, Deuschle M, Gutberlet M, *et al.* (2015): Adrenal gland volume, intra-abdominal and pericardial adipose tissue in major depressive disorder. *Psychoneuroendocrinology* 58: 1–8.

11. Santana MM, Rosmaninho-Salgado J, Cortez V, Pereira FC, Kaster MP, Aveleira CA, *et al.* (2015): Impaired adrenal medullary function in a mouse model of depression induced by unpredictable chronic stress. *Eur Neuropsychopharmacol* 25: 1753–1766.

12. Varghese FP, Brown ES (2001): The Hypothalamic-Pituitary-Adrenal Axis in Major Depressive Disorder: A Brief Primer for Primary Care Physicians. *Prim Care Companion J Clin Psychiatry* 3: 151–155.

13. Kunugi H, Hori H, Adachi N, Numakawa T (2010): Interface between hypothalamic-pituitary-adrenal axis and brain-derived neurotrophic factor in depression. *Psychiatry Clin Neurosci* 64: 447–459.

14. Kessing LV, Willer IS, Knorr U (2011): Volume of the adrenal and pituitary glands in depression. *Psychoneuroendocrinology* 36: 19–27.

15. Delvecchio G, Altamura AC, Soares JC, Brambilla P (2017): Pituitary gland in Bipolar Disorder and Major Depression: Evidence from structural MRI studies: Special Section on "Translational and Neuroscience Studies in Affective Disorders". *J Affect Disord* 218: 446–450.

16. Pedersen C, Leserman J, Garcia N, Stansbury M, Meltzer-Brody S, Johnson J (2016): Late pregnancy thyroid-binding globulin predicts perinatal depression. *Psychoneuroendocrinology* 65: 84–93.

17. Ittermann T, Völzke H, Baumeister SE, Appel K, Grabe HJ (2015): Diagnosed thyroid disorders are associated with depression and anxiety. *Soc Psychiatry Psychiatr Epidemiol* 50: 1417–1425.

18. Duval F, Mokrani M-C, Erb A, Gonzalez Lopera F, Alexa C, Proudnikova X, Butucaru I (2015): Chronobiological hypothalamic–pituitary–thyroid axis status and antidepressant outcome in major depression. *Psychoneuroendocrinology* 59: 71–80.

19. Delitala AP, Terracciano A, Fiorillo E, Orrù V, Schlessinger D, Cucca F (2016): Depressive symptoms, thyroid hormone and autoimmunity in a population-based cohort from Sardinia. *J Affect Disord* 191: 82–87.

20. Zhou Y, Wang X, Zhao Y, Liu A, Zhao T, Zhang Y, *et al.* (2017): Elevated Thyroid Peroxidase Antibody Increases Risk of Post-partum Depression by Decreasing Prefrontal Cortex BDNF and 5-HT Levels in Mice. *Front Cell Neurosci* 10. https://doi.org/10.3389/fncel.2016.00307

21. Dudbridge F, Gusnanto A (2008): Estimation of significance thresholds for genomewide association scans. *Genet Epidemiol* 32: 227–234.

22. Giambartolomei C, Vukcevic D, Schadt EE, Franke L, Hingorani AD, Wallace C, Plagnol V (2014): Bayesian Test for Colocalisation between Pairs of Genetic Association Studies Using Summary Statistics ((S. M. Williams, editor)). *PLoS Genet* 10: e1004383.

23. Ziyatdinov A, Vázquez-Santiago M, Brunel H, Martinez-Perez A, Aschard H, Soria JM (2018): lme4qtl: linear mixed models with flexible covariance structure for genetic studies of related individuals. *BMC Bioinformatics* 19: 68.

24. Wray NR, Ripke S, Mattheisen M, Trzaskowski M, Byrne EM, Abdellaoui A, *et al.* (2018): Genome-wide association analyses identify 44 risk variants and refine the genetic architecture of major depression. *Nat Genet* 50: 668.

25. Gaspar HA, Gerring Z, Hübel C, Middeldorp CM, Derks EM, Breen G (2019): Using genetic drug-target networks to develop new drug hypotheses for major depressive disorder. *Transl Psychiatry* 9: 117.

26. Gerring ZF, Gamazon ER, Derks EM, Consortium for the MDDWG of the PG (2019): A gene co-expression network-based analysis of multiple brain tissues reveals novel genes and molecular pathways underlying major depression. *PLOS Genet* 15: e1008245.
